# ct-DNA Binding and Antibacterial Activity of Octahedral Titanium (IV) Heteroleptic (Benzoylacetone and Hydroxamic Acids) Complexes

**DOI:** 10.1155/2016/2361214

**Published:** 2016-03-16

**Authors:** Raj Kaushal, Sheetal Thakur, Kiran Nehra

**Affiliations:** ^1^Department of Chemistry, National Institute of Technology, Hamirpur, Himachal Pradesh 177005, India; ^2^Department of Biotechnology, Deenbandhu Chhotu Ram University of Science and Technology, Murthal, Sonepat, Haryana 131039, India

## Abstract

Five structurally related titanium (IV) heteroleptic complexes, [TiCl_2_(bzac)(L^1–4^)] and [TiCl_3_(bzac)(HL^5^)]; bzac = benzoylacetonate; L^1–5^ = benzohydroximate (L^1^), salicylhydroximate (L^2^), acetohydroximate (L^3^), hydroxyurea (L^4^), and N-benzoyl-N-phenyl hydroxylamine (L^5^), were used for the assessment of their antibacterial activities against ten pathogenic bacterial strains. The titanium (IV) complexes (**1**–**5**) demonstrated significant level of antibacterial properties as measured using agar well diffusion method. UV-Vis absorption spectroscopic technique was applied, to get a better insight into the nature of binding between titanium (IV) complexes with calf thymus DNA (ct-DNA). On the basis of the results of UV-Vis absorption spectroscopy, the interaction between ct-DNA and the titanium (IV) complexes is likely to occur through the same mode. Results indicated that titanium (IV) complex can bind to calf thymus DNA (ct-DNA) via an intercalative mode. The intrinsic binding constant (*K*_*b*_) was calculated by absorption spectra by using Benesi-Hildebrand equation. Further, Gibbs free energy was also calculated for all the complexes.

## 1. Introduction

Nowadays, metal complexes have been used as powerful chemotherapeutic agents to cure variety of diseases due to their ease in altering ligand exchange reactions rates, redox properties, oxidation states, and coordination number in order to enhance their efficacy with less side effects. Metal complexes have shown powerful antimicrobial activities and are used in different treatments like silver bandages for treatment of burns, zinc antiseptic creams, bismuth drugs for the treatment of ulcers, organotin drugs as antileishmanial agents, and metal clusters as HIV drugs [1–3]. Cisplatin and its analogues, namely, carboplatin, oxaliplatin, tetraplatin, and satraplatin, have shown remarkable antiproliferative activity. However, due to their drug toxicity and resistance to cell lines, their utilization for a broader range of diseases is limited [4]. Recent research showed that titanium complexes have interesting cytotoxic properties because of their less toxicity, different oxidation states (4^+^, 3^+^, and 2^+^) and capability of binding to DNA. Most of the complexes, that is, derivatives of either Cp_2_TiCl_2_ or [Ti(bzac)_2_(OEt)_2_]; Hbzac = [phenylbutane-1,3-dione], are investigated for biological activity [5]. All these complexes showed very promising results in preclinical as well as clinical studies and attracted a high attention in medical research because of their comparative activity and lower cytotoxicity [6, 7].

The interaction and reaction of transition metal complexes with DNA have long been intensively investigated in relation to applications in the fields of molecular biology, biotechnology, and medicine [8]. Generally, DNA plays an important role in the life process since it contains all the genetic information for cellular function. However, DNA molecules are prone to damage under various conditions like interactions with some molecules. This damage may lead to various pathological changes in living organisms. The binding interaction of transition metal complexes to DNA is of interest for both therapeutic and scientific reasons [9, 10].

These transition metal complexes are known to bind to DNA via both covalent and noncovalent interactions. In covalent binding the labile ligand of the complexes is replaced by a nitrogen base of DNA such as guanine N7. On the other hand, the noncovalent DNA interactions include intercalative, electrostatic, and groove (surface) binding of cationic metal complexes along the outside of DNA helix, along major or minor groove. Interest in the fashion of metal complex binding to DNA has been motivated not only by a desire to understand the basics of these interaction modes but also by the development of metal complexes into anti-inflammatory, antifungal, antibacterial, or anticancer reagents. Hence, much of the attention has been targeted on the design of metal-based complexes, which can bind to DNA.

In continuation of our work on development of new transition metals which bind to ct-DNA, herein we report the ct-DNA binding studies of some selected titanium (IV) heteroleptic ligands complexes by the absorption titration method. Furthermore, their antibacterial activities were tested against certain human pathogenic organisms by agar well diffusion method. The minimum inhibitory concentration (MIC) was also obtained using the macrodilution test.

## 2. Materials and Methods

Titanium tetrachloride, benzoylacetone, benzohydroxamic acid, salicylhydroxamic acid, acetohydroxamic acid, hydroxyurea, and N-phenyl n-benzyl hydroxamic acid were obtained from Aldrich and Merck products and used as such after checking their melting point/boiling point. UV-Vis spectroscopy was carried out on a Perkin-Elmer Lambda-1600 spectrophotometer.

### 2.1. *In Vitro* Antibacterial Activity

Antibacterial activity was determined by the agar well diffusion method [11]. The investigated microorganisms were* E. aerogenes* MTCC 6128,* Micrococcus luteus* MTCC 1809,* Staphylococcus aureus* MTCC 3160,* Staphylococcus epidermidis* MTCC 3086,* Aeromonas hydrophila* MTCC 1739,* Alcaligenes faecalis* MTCC 126,* Shigella sonnei* MTCC 2957,* Klebsiella pneumoniae* MTCC 3384,* Pseudomonas aeruginosa* MTCC 1035, and* Salmonella typhimurium* MTCC 1253. The complexes were dissolved in DMSO solvent to obtain a final concentration of 1 mg/1 mL. A loop full of the given test strain was inoculated in 25 mL of N-broth (nutrient broth) and incubated for 24 h in an incubator at 37°C in order to activate the bacterial strain. 28–30 mL of the nutrient agar media was added into a 100 mm diameter Petri plate. Inoculation was done by the pour-plate technique. 0.1 mL of the activated strain was inoculated into the media when it reached a temperature of 40–45°C. The complete procedure of the plate preparation was done in a laminar airflow to maintain strict sterile and aseptic condition. The medium was allowed to solidify. After solidification of the media, a well was made in the plates with the help of a cup-borer (0.85 cm), which was then filled with one of the test sample solutions. Controls were run for each bacterial strain, where DMSO was inoculated into the well. The plates were incubated for 24 h at 37°C. The inhibition zone formed by these compounds against the particular test bacterial strain determined the antibacterial activities of the synthesized complexes. The mean value obtained for two individual replicates was used to calculate the zone of growth inhibition of each sample.

### 2.2. UV-Vis Spectroscopic Analysis for Calf Thymus DNA Binding

UV-Vis spectrophotometry was used to study the interactions of newly synthesized titanium (IV) complexes with ct-DNA in double distilled water containing tris-HCl (1 M, pH 7.4). The concentration of solution of ct-DNA used for binding studies was determined spectrophotometrically at 260 nm (*ε* = 6600 M^−1^ cm^−1^) [12]. A solution of ct-DNA in the buffer gave a ratio of UV absorbance at 260 and 280 nm (*A*_260_/*A*_280_) of 1.8, indicating that the ct-DNA is sufficiently free of protein [13, 14]. The ct-DNA concentration, that is, 0.19, 0.18, and 0.17 mM, was selected for complex (**1**). The varying concentrations of ct-DNA for complex (**2**) were 0.162, 0.169, 0.19, 0.21, and 0.23 mM. For complex (**3**), the conc. varied in 0.35, 0.39, 0.45, and 0.51 mM of ct-DNA. The concentrations of ct-DNA for complex (**4**) were 0.19, 0.20, and 0.23 mM. The binding experiments were carried out by titrating increasing concentrations of ct-DNA (0.36, 0.31, 0.29, and 0.15 mM) against 5 *μ*L of 1 mM for complex (**5**). All the solutions were prepared in tris-HCl buffer solution. Upon these dilution of ct-DNA the 5 *μ*L of 1 mM solution of investigated titanium (IV) heteroleptic complexes was added with micropipette by making the volume constant up to 2 mL. First of all, *λ*_max_ and absorbance of pure ct-DNA, serial dilution without complexes and with titanium (IV) complexes in buffer solutions were recorded. 2 mL of each solution of ct-DNA and titanium (IV) complexes were mixed together and their *λ*_max_ and absorbance values recorded. The absorption spectra were recorded after each addition of different concentrations of ct-DNA solution (2.0 mL).

## 3. Results and Discussion

Synthesis of titanium complexes with the general formulas [TiCl_2_(bzac)(L^1–4^)] (**1**–**4**) and [TiCl_3_(bzac)(HL^5^)] (**5**) was carried out in two steps. In step 1, titanium tetrachloride was reacted with benzoylacetone (bzac) (O, O) bidentate ligand in 1 : 1 molar ratio under continuous stirring and refluxing by using methanol as a solvent. There was evolution of HCl gas which was observed during the course of reaction. The presence of HCl gas was ensured by the evolution of dense white fume after putting the ammonia dipped rod on the mouth of round bottom flask. The reaction mixture was stirred for 2 hr followed by the refluxing up to the completion of reaction.

**Figure 1:**
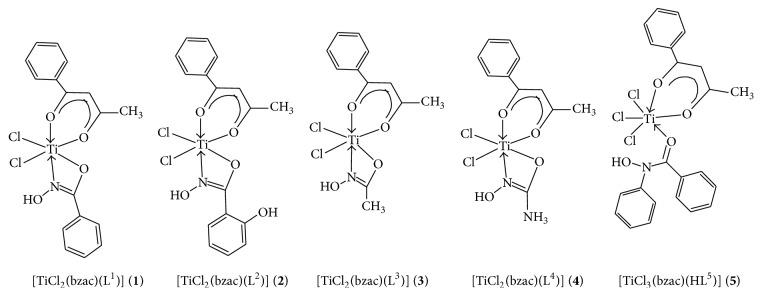
Proposed structures of titanium (IV) heteroleptic complexes (**1**–**5**).

In the next step, to the above reaction mixture, the respective hydroxamic acid ligands solution in methanol was added dropwise in 1 : 1 molar ratio with continuous stirring. Further, the reaction mixture refluxed for 16 h till the complete evolution of HCl gas. The proposed structure of synthesized titanium (IV) complexes has been depicted in Figure 1.

### 3.1. *In Vitro* Antibacterial Activity

*In vitro*, biological screening of the titanium (IV) complexes (**1**–**5**) was tested against ten pathogenic bacterial strains, that is,* E. aerogenes* MTCC 6128,* Micrococcus luteus* MTCC 1809,* Staphylococcus aureus* MTCC 3160,* Staphylococcus epidermidis* MTCC 3086,* Aeromonas hydrophila* MTCC 1739,* Alcaligenes faecalis* MTCC 126,* Shigella sonnei* MTCC 2957,* Klebsiella pneumoniae* MTCC 3384,* Pseudomonas aeruginosa* MTCC 1035, and* Salmonella typhimurium* MTCC 1253. The zone of inhibition diameter (mm) of the novel investigated titanium (IV) complexes against the growth of organisms was summarized in Table 1.

On the basis of the result, it was found that complex [TiCl_2_(bzac)(L^1^)] (**1**) was more potent against* S. sonnei* with 13.5 mm diameter and [TiCl_2_(bzac)(L^2^)] (**2**) is more active against* A*.* hydrophila* with 18 mm zone inhibition diameter among all the bacterial strains. Complex [TiCl_2_(bzac)(L^3^)] (**3**) was found to be more active against* E. aerogenes*,* S. epidermidis*, and* S. sonnei* with 13 mm, 13 mm, and 14.5 mm zone inhibition diameter, respectively. Complex [TiCl_2_(bzac)(L^4^)] (**4**) is more potent against* S. typhimurium*,* E. aerogenes*,* S. epidermidis*,* S. aureus*, and* M. luteus* with 13 mm, 13 mm, 15 mm, 15 mm, and 16.5 mm zone inhibition diameters, respectively, and complexes [TiCl_3_(bzac)(HL^5^)] (**5**) is more effective against* P. aeruginosa*,* S. aureus*, and* A. faecalis* with 17 mm, 16 mm, and 14 mm zone inhibition diameters, respectively.

A comparative study of ligands and their titanium (IV) complexes showed that complexes exhibit higher antibacterial activity than their parent ligands. Such increased activity of metal chelate can be explained on the basis of the overtone concept and chelation theory. According to the overtone concept of cell permeability the lipid membrane that surrounds the cell favors the passage of only lipid soluble materials in which liposolubility is an important factor that controls the antimicrobial activity. On chelating, the polarity of the metal ion will be reduced to a greater extent due to overlap of ligand orbital and partial sharing of the positive charge of the metal ion with donor groups. Further, it increases the delocalization of *π*-electrons over the whole chelate ring and enhances the lipophilicity of complexes [15, 16]. This increased lipophilicity enhances the penetration of complexes into the lipid membranes and blocks the metal binding sites in enzymes of microorganisms. These complexes also disturb the respiration process of the cell and thus block the synthesis of proteins, which restricts further growth of the organism.

**Figure 2:**
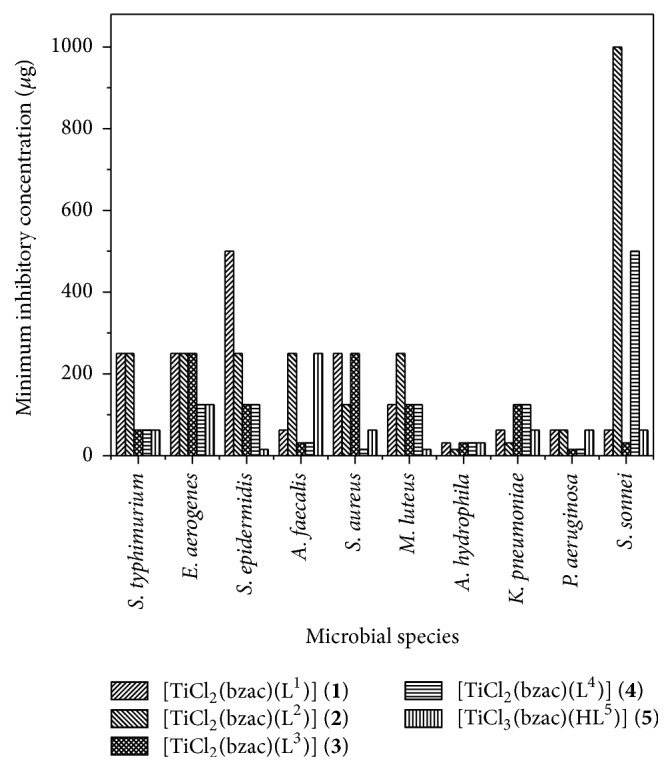
Bar graph of minimum inhibitory concentration (MIC) of selected titanium heteroleptic (IV) complexes (**1**–**5**) in *μ*g versus pathogenic bacterial strains.

From the MIC values determination as shown in (Figure 2), it was found that, from the complexes, [TiCl_3_(bzac)(L^5^)] (**5**) was more potent against* P. aeruginosa* and* S. aureus* with MIC value of 15.6 *μ*g and [TiCl_2_(bzac)(L^4^)] (**4**) was more potent against* M. luteus* and* S. epidermidis* with 15.6 *μ*g dose concentration. [TiCl_2_(bzac)(L^3^)] (**3**) and [TiCl_2_(bzac)(L^2^)] (**2**) were more potent against* P. aeruginosa *and* A. hydrophila*, respectively, with 15.6 *μ*g concentration of respected titanium (IV) complexes. [TiCl_2_(bzac)(L^1^)] (**1**) is more potent against* P. aeruginosa* with 62.5 *μ*g MIC value compared to the other bacterial strains, respectively. Ampicillin as a positive control was found to be most effective against* S. typhimurium*,* E. aerogenes*, and* S. epidermidis* and least effective against* P. aeruginosa* bacterial strain among all the pathogenic bacterial strains. As reported earlier, the antibacterial activities of titanium complexes will be enhanced when it is coordinated by oxygen atom of bidentate ligand instead of nitrogen atom [17, 18]. Therefore, antibacterial activity of complexes depends upon metal as well as properties of ligands.

In titanium (IV) complexes, titanium metal acquires *d* ^0^ electronic configuration so it acts as hard Lewis acid and has tendency to coordinate with oxygen, nitrogen, and sulphur atom. So tendency to form the complexes is Ti-O > Ti-N > Ti-Cl > Ti-P > Ti-S. As zero ligand field stabilization energy of titanium (IV) complexes (**1**–**5**), these form labile complexes which allow rapid exchange with a new ligand from within the biological environment; that is, protein and nucleic acid offer many potential metal-ligand sites such as sulphur, nitrogen, and oxygen atoms [19]. As earlier reported free metal ion was found not to be less active than their complexes because complexation of metal ion prevents its interaction with a host of possible substance in the cell or in a cell membrane of microorganism such as oxygen, thiol, or other electron rich centers, thus precluding its biocide activity.

### 3.2. ct-DNA Binding Studies through Electronic Absorption Titration

UV-Vis absorption spectroscopy is one of the most effective techniques to study the interaction between nucleic acids and metal complexes [18, 19]. Molecules containing aromatic groups and nitrogen, oxygen, and sulphur donor atom can interact with ct-DNA [20, 21]. Therefore, the interaction can be investigated based on comparison of absorption spectra before and after the reaction.

**Figure 3:**
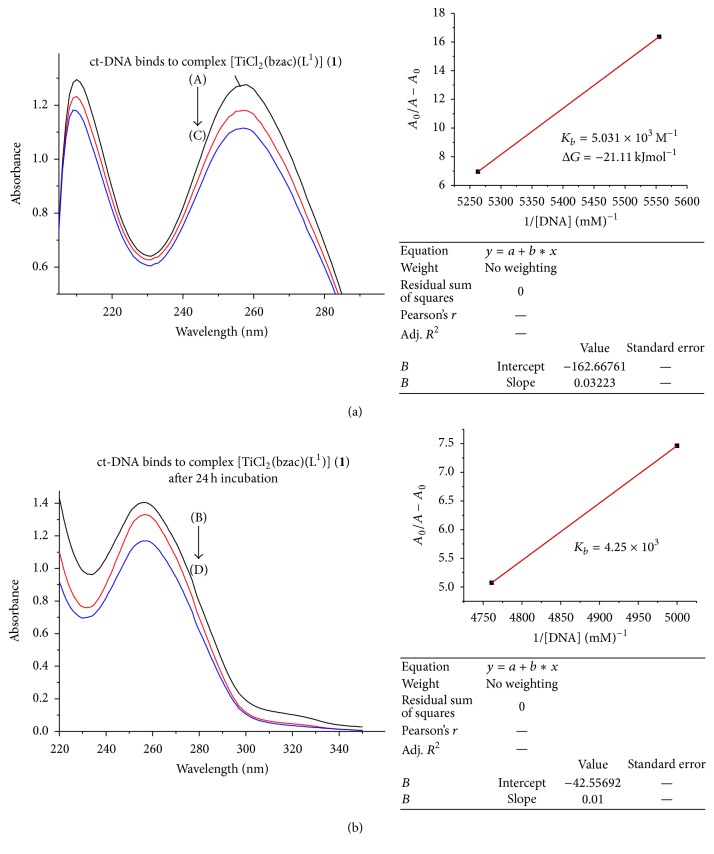
(a) Absorption spectrum of ct-DNA without complex (**1**) having concentration 0.17 mM and complex (**1**) adduct with ct-DNA with varying concentration of ct-DNA 0.18 mM (B) and 0.19 mM (C). The arrow indicated the increasing conc. of ct-DNA. The graph represents the plot of *A*_0_/(*A* − *A*_0_) versus 1/[DNA] (mM)^−1^ for the calculation of binding constant (*K*_*b*_). (b) Absorption spectrum of ct-DNA with complex (**1**) after 24 h incubation.

**Figure 4 fig4:**
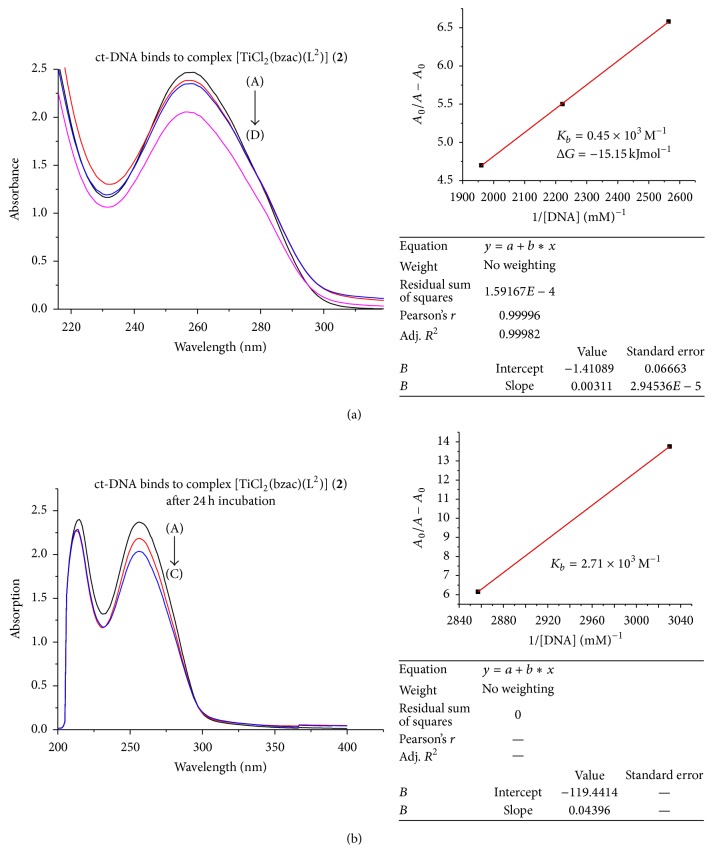
(a) Absorption spectrum of ct-DNA without complex (**2**) having concentration 0.35 mM (A) and complex (**3**) adduct with ct-DNA with varying concentration of ct-DNA 0.39 mM (B), 0.45 (C), and 0.51 mM (D). The arrow indicated the increasing conc. of ct-DNA. The graph represents the plot of *A*_0_/(*A* − *A*_0_) versus 1/[DNA] (mM)^−1^ for the calculation of binding constant (*K*_*b*_). (b) Absorption spectrum of ct-DNA with complex (**2**) after 24 h incubation.

**Figure 5 fig5:**
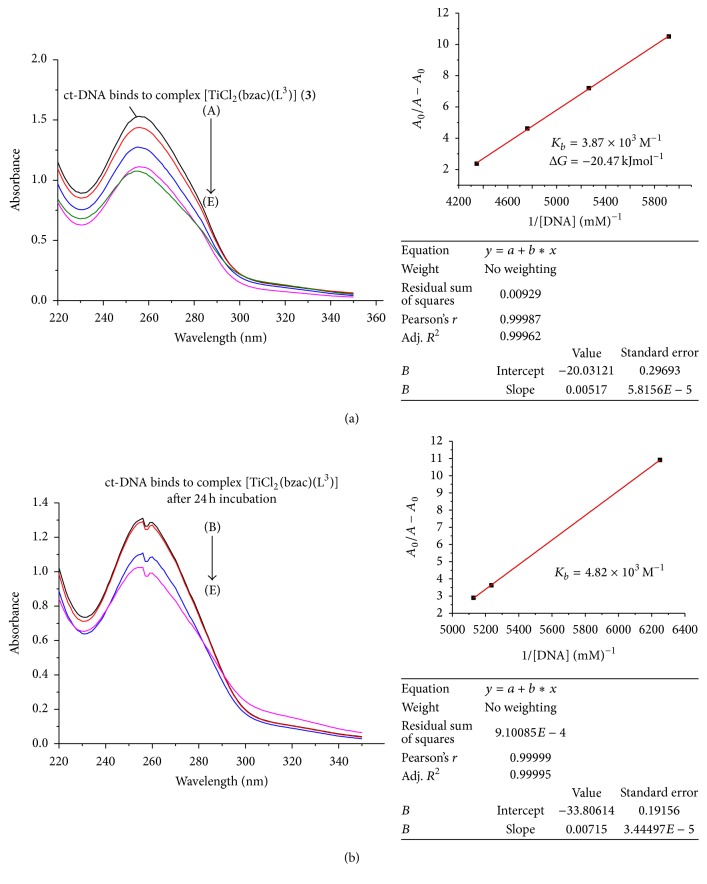
(a) Absorption spectrum of ct-DNA without complex (**3**) having concentration 0.162 mM (A) and complex (**3**) adduct with ct-DNA with varying concentration of ct-DNA 0.169 mM (B), 0.19 mM (C), 0.21 mM (D), and 0.23 mM (E). The arrow indicated the increasing conc. of ct-DNA. The graph represents the plot of *A*_0_/(*A* − *A*_0_) versus 1/[DNA] (mM)^−1^ for the calculation of binding constant (*K*_*b*_). (b) Absorption spectrum of ct-DNA with complex (**3**) after 24 h incubation.

**Figure 6 fig6:**
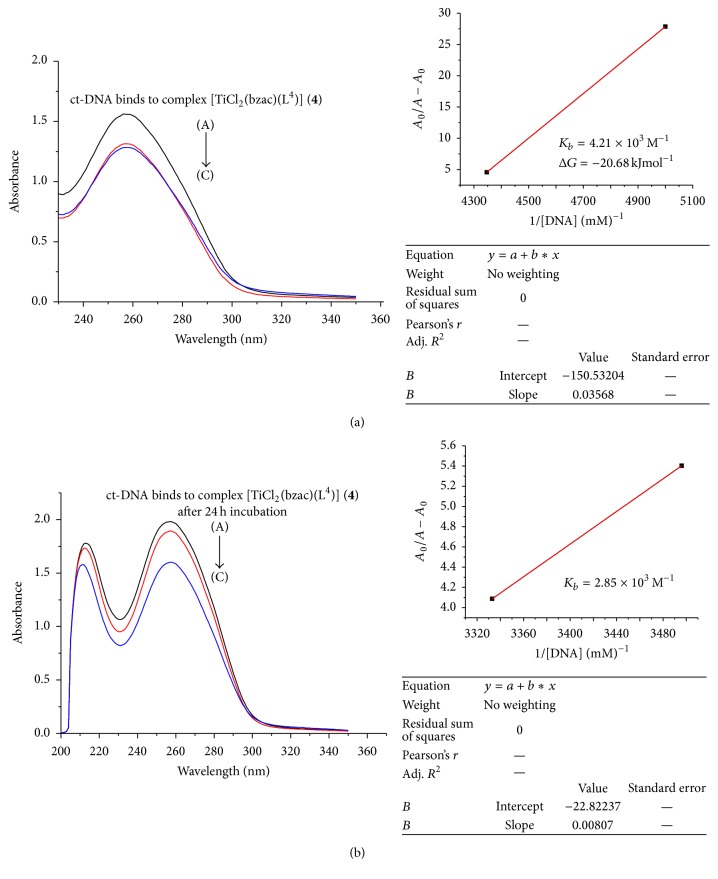
(a) Absorption spectrum of ct-DNA without complex (**4**) having concentration 0.19 mM (A) and complex (**4**) adduct with ct-DNA with varying concentration of ct-DNA 0.20 mM (B) and 0.23 mM (C). The arrow indicated the increasing concentration of ct-DNA. The graph represents the plot of *A*_0_/(*A* − *A*_0_) versus 1/[DNA] (mM)^−1^ for the calculation of binding constant (*K*_*b*_). (b) Absorption spectrum of ct-DNA with complex (**4**) after 24 h incubation.

**Figure 7:**
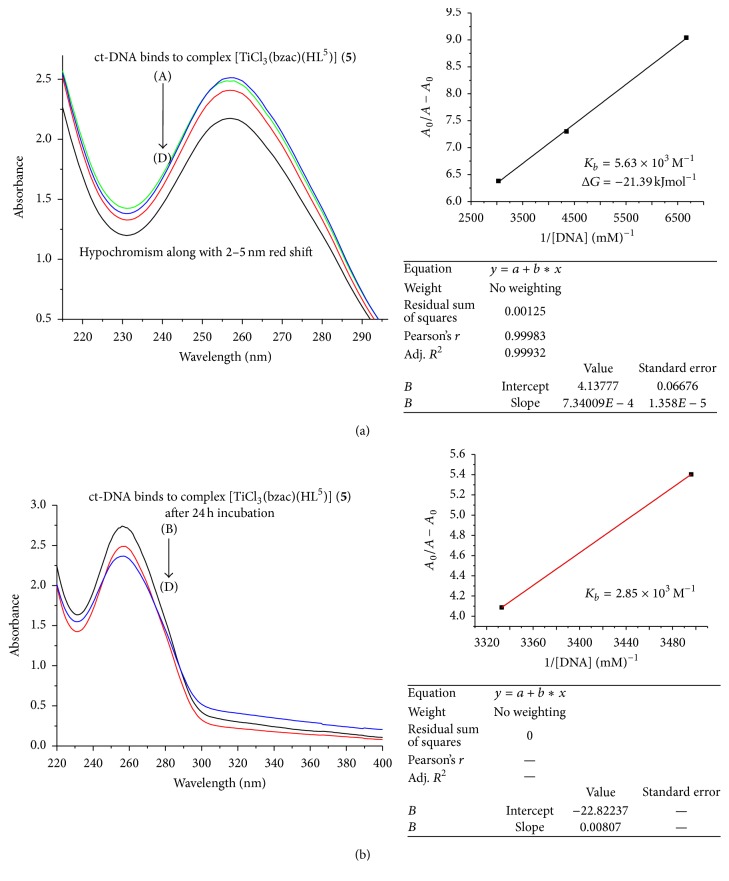
(a) Absorption spectrum of ct-DNA without complex (**5**) having concentration 0.15 mM and complex (**5**) adduct with ct-DNA with varying concentration of ct-DNA 0.29 mM (B), 0.31 (C), and 0.36 mM (D). The arrow indicated the increasing concentration of ct-DNA. The graph represents the plot of *A*_0_/(*A* − *A*_0_) versus 1/[DNA] (mM)^−1^ for the calculation of binding constant (*K*_*b*_). (b) Absorption spectrum of ct-DNA with complex (**5**) after 24 h incubation.

In the UV region (Figures 3(a) [Fig fig4] [Fig fig5] [Fig fig6]–7(a)), the ct-DNA without compound exhibits intense absorption bands at 256 nm, which can be attributed to *π*-*π* ^*∗*^ intraligand transitions. Obvious hypochromism was observed upon increasing the concentration of ct-DNA. To explore the ct-DNA-binding mode of newly synthesized titanium (IV) heteroleptic complexes, 5 *μ*L of 1 mM of each complex was added and incubated for 24 h in presence of an increasing concentration of ct-DNA (0.1–0.40 mM). However, as a function of ct-DNA concentration, the intensities of the maximum absorption peaks at 256 nm were demonstrated as a gradual reduction with red shift.

As shown in Figures 3(a)–7(a), the addition of ct-DNA induces a hypochromic effect, accompanying red shifts (bathochromism) of 2–5 nm. During intercalating, the *π* ^*∗*^ orbital of intercalated metal complex was coupled with the *π* orbital of base pairs, thus decreasing the *π*-*π* ^*∗*^ transition energy, further resulting in bathochromism. On the other hand, the coupling of a *π* orbital with partially filled electrons decreases the transition probabilities and hence results in hypochromic shift. Since hypochromism due to *π*-*π* ^*∗*^ stacking interaction may appear in the case of the intercalative binding mode, while bathochromism may be observed when the ct-DNA duplex is stabilized, the observed hypochromism and bathochromic shift might be attributed to stacking interaction between the chromophores of newly synthesized titanium (IV) complexes and ct-DNA base pairs, which were consistent with the intercalative binding mode [22]. Based upon the variation in absorbance, the intrinsic binding constant of the complexes with ct-DNA was determined according to Benesi-Hildebrand equation [23]:

(1) $$\begin{array}{c} \frac{A_{0}}{A-A_{0}}=\frac{\varepsilon_{G}}{\varepsilon_{H-G}-\varepsilon_{G}}+\frac{\varepsilon_{G}}{\varepsilon_{H-G}-\varepsilon_{G}}\times \frac{1}{K_{b}\left[DNA\right]}, \end{array}$$

where *K*_*b*_ is the association/binding constant, *A*_0_ and *A* are the absorbance of the ct-DNA without complex and ct-DNA with complex, respectively, and *ε*_*G*_ and *ε*_*H*−*G*_ are the absorption coefficients of the ct-DNA without complex and the ct-DNA with complex, respectively. The association constants were obtained from the intercept-to-slope ratios of *A*_0_/(*A* − *A*_0_) versus 1/[DNA] plots.

The binding constant for each complex (**1**–**5**) was found to be 5.03 × 10^3^, 0.45 × 10^3^, 3.82 × 10^3^, 4.2 × 10^3^, and 5.63 × 10^3^, respectively, as shown in Table 2. Out of these complexes, complex (**5**) has the highest and complex (**2**) has lowest binding constant. So upon the above discussion, it can be concluded that complex (**5**) was a stronger intercalator for ct-DNA. So it might be used as a potent anticancerous agent because it was proposed that metal complexes interact with nitrogenous bases of nucleotides of nucleic acid and inhibit the cell division by interfering with the replication and transcription. The complexes may also affect the multienzyme complexes responsible for replication and transcription of ct-DNA, thus causing a stop of proliferation of the cells [24].

By comparing the binding complexes of titanium (IV) complexes, it was found that complex (**1**) has 5.03 × 10^3^ M^−1^ binding constant and after 24 hr incubation this value decreased as shown in Figures 3(b)–7(b) and it has 4.25 × 10^3^ M^−1^ indicating that, upon incubation, the ct-DNA-complex (**1**) adduct gets destabilized due to destabilization of ct-DNA. For complexes (**2**) and (**3**), the reverse trend was observed as compared to complex (**1**), indicating that, with passage of time, ct-DNA-complex gets stabilized. But the same trend was observed for complexes (**3**), (**4**), and (**5**) as complex (**1**) showed that ct-DNA-complex adduct gets destabilized after incubation.

The Gibbs free energy (Δ*G*) was determined from the equation:

(2) $$\begin{array}{c} \Delta G=-RT\ln K_{b}, \end{array}$$

where *R* is the general gas constant (8.314 JK^−1^mol^−1^) and *T* is the temperature (298 K). The Gibbs free energy (Δ*G*) for all the selected titanium (IV) complexes showed that, except for complex (**2**), left titanium (IV) complexes have decreased Δ*G* count compared to that before incubation. This observation indicated less feasibility of reaction after 24 hr incubation between adduct of ct-DNA and selected titanium (IV) complexes. But complex (**2**) acquired Δ*G* (−19.59 kJ mol^−1^) after 24 hr incubation, which was higher than that before incubation, Δ*G* value −15.15 kJ mol^−1^, showing more feasibility of ct-DNA and complex (**2**) adduct formation.

## 4. Conclusion

From the above discussion, it can be concluded that inhibitory potencies of the selected titanium (IV) complexes (**1**–**5**) are found to have high antibacterial activity compared to parent ligand. The aromaticity, electron donation, bulky nature of ligands substituents played an important role in enhancing the antibacterial activity of titanium (IV) metal complexes. On the basis of zone inhibition diameter and MIC value, it was concluded that selected titanium (IV) complexes (**1**–**5**) were more potent against* A*.* hydrophila*,* S. aureus*,* E. aerogenes*,* S. sonnei*,* P. aeruginosa*,* S. typhimurium*,* S. epidermidis*, and* M. luteus* than other bacterial strains. Studies on ct-DNA binding to titanium (IV) complexes indicated that all these selected titanium (IV) complexes bind with ct-DNA through intercalation mode. Out of these, complex (**5**) acquired the highest 5.6 × 10^3^ M^−1^ binding constant. Further, ct-DNA complex (**5**) was found to be more feasible with 21.39 kJ mol^−1^Δ*G* value.

## Figures and Tables

**Table 1:** Zone inhibition diameter (mm) of selected titanium (IV) heteroleptic complexes (**1–5**) and corresponding ligands (in parentheses) against pathogenic bacterial strains.

Sr. number	Microbial species	Zone inhibition diameter (mm)					
		Complex (ligand^*∗*^)					
		(1) (L^1^)	(2) (L^2^)	(3) (L^3^)	(4) (L^4^)	(5) (L^5^)	Ampicillin
1	*S. typhimurium*	10 (9.5)	10 (9)	11.5 (10)	13 (11.5)	11 (9.5)	16
2	*E. aerogenes*	10 (9.5)	10 (8)	13 (11)	13 (12)	12 (10.5)	15
3	*S. epidermidis*	8 (8)	10 (8.5)	13 (11.5)	15 (12.5)	10 (9.5)	16
4	*A. faecalis*	10.5 (9)	11.5 (9.5)	12.5 (10.5)	10.5 (9.5)	14 (12.5)	10
5	*S. aureus*	10 (8.5)	10.5 (9.5)	10 (8.5)	15 (12.5)	16 (12.5)	12
6	*M. luteus*	11 (9.5)	10 (9)	10.5 (9.5)	16.5 (13.5)	10 (8.5)	10
7	*A. hydrophila*	12 (10.5)	18 (13.5)	12 (10.5)	12 (10.5)	12 (11)	13
8	*K. pneumoniae*	11 (9.5)	14 (12.5)	10 (8.5)	12 (10.5)	10 (9)	11
9	*P. aeruginosa*	12 (11)	12 (10.5)	16 (14.5)	12 (10.5)	17 (15.5)	8
10	*S. sonnei*	13.5 (11.5)	7.5 (7)	14.5 (12.5)	11 (9.5)	8 (7.5)	13

^*∗*^Benzohydroxamic acid (L^1^), salicylhydroxamic acid (L^2^), acetohydroxamic acid (L^3^), hydroxyurea (L^4^), and N-benzoyl-N-phenyl hydroxylamine (L^5^).

**Table 2:** Binding constant and Gibbs free energy data for titanium (IV) heteroleptic complexes (**1–5**).

Sr. number	Titanium (IV) complexes	Binding constant (*K*_*b*_) (mol^−1^)	Δ*G* (kJ mol^−1^)
1	[TiCl_2_(bzac)(L^1^)] (1)	5.03 × 10^3^	−21.11
2	[TiCl_2_(bzac)(L^2^)] (2)	0.45 × 10^3^	−15.15
3	[TiCl_2_(bzac)(L^3^)] (3)	3.87 × 10^3^	−20.47
4	[TiCl_2_(bzac)(L^4^)] (4)	4.21 × 10^3^	−20.68
5	[TiCl_3_(bzac)(HL^5^)] (5)	5.63 × 10^3^	−21.39
